# Negative pressure wound therapy for perianal necrotizing fasciitis: case report and literature review

**DOI:** 10.3389/fsurg.2026.1747876

**Published:** 2026-02-24

**Authors:** Hao Ma, Bei Zhang, Yahong Xue, Qingrui Liu, Jiahui Chen, Pei Wang, Hao Ge

**Affiliations:** 1 Department of Anorectal Center, Nanjing Hospital of Chinese Medicine Affiliated to Nanjing University of Chinese Medicine, Nanjing, China; 2 Nanjing University of Chinese Medicine, Nanjing, China

**Keywords:** fecal diversion, fournier's gangrene, negative pressure wound therapy (NPWT), perianal necrotizing fasciitis, radical debridement, vacuum-assisted closure (VAC)

## Abstract

Perianal necrotizing fasciitis (PNF) is a life-threatening soft tissue infection with high mortality. Despite the established role of radical debridement, outcomes are often hindered by late diagnosis and suboptimal postoperative care. While negative pressure wound therapy (NPWT) promotes complex wound healing, its synergistic application with radical debridement in PNF requires further validation. We report a case of extensive PNF that demonstrated the limitations of traditional interventions. By utilizing an integrated strategy of extended-incision radical debridement and early NPWT, we achieved rapid infection control and accelerated wound healing. This case suggests that such a combined technical framework can overcome clinical challenges and significantly improve PNF prognosis.

## 1 Introduction

Fournier's gangrene (FG), a subset of perianal necrotizing fasciitis (PNF), is a fulminant and rapidly progressive necrotizing soft tissue infection primarily involving the perianal, perineal, and genital regions. Characterized by its aggressive spread along fascial planes, FG often precipitates systemic sepsis and multi-organ failure if not managed with immediate intervention. Despite its relatively low incidence, the associated mortality remains high, underscoring the necessity of early clinical recognition and aggressive surgical debridement (1). While radical debridement remains the cornerstone of treatment, conventional techniques—often involving open drainage and frequent dressing changes—frequently encounter obstacles such as complex wound geometry, difficulty in exudate management, and protracted healing times (2). Negative pressure wound therapy (NPWT) has emerged as a potent adjunctive modality, offering distinct advantages in managing infected wounds by optimizing the wound microenvironment and stimulating granulation tissue (3). However, a standardized, systematic protocol for integrating NPWT with specific debridement strategies for FG has yet to be fully elucidated.This article presents a case of extensive Fournier's gangrene where the clinical course was complicated by delayed initial diagnosis. By employing a synergistic approach of extended radical debridement coupled with early-stage NPWT for continuous drainage, we achieved rapid infection control and accelerated tissue repair. This case report aims to explore the therapeutic efficacy of this integrated framework, providing a clinical reference for the management of such complex and life-threatening infections.

## 2 Case report

### 2.1 Clinical history and initial presentation

A 63-year-old male with a 30-year history of urinary and fecal incontinence following a fall from height presented with a one-week history of rapidly progressing perianal symptoms. On October 1, he developed a high-grade fever (peak 39.1 °C), followed by the emergence of a painful, erythematous, and edematous perianal mass. Despite transient improvement with antibiotics at a local clinic, the mass expanded rapidly, exhibiting central liquefactive necrosis. By day 7, the erythema and swelling had disseminated to the buttocks, lower back, abdominal wall, and left lower extremity. The mass subsequently ruptured, discharging malodorous, grayish-black purulent exudate. Despite multiple consultations, the diagnosis remained elusive, and empiric penicillin therapy proved ineffective.

### 2.2 Admission and diagnostic assessment

Upon transfer to our center on October 8, the patient was in a state of septic shock and altered mental status, presenting with delirium, chest tightness, and tachypnea. Vital signs revealed a blood pressure of 95/45 mmHg and a temperature of 36.6 °C. Physical examination demonstrated extensive erythema and swelling with palpable crepitus extending from the fourth lumbar vertebra superiorly to the left ankle inferiorly, including the buttocks and left inguinal region. A 2 × 2 cm necrotic area was noted at the 3–6 o’clock perianal position with active purulent drainage.

**Figure 1:**
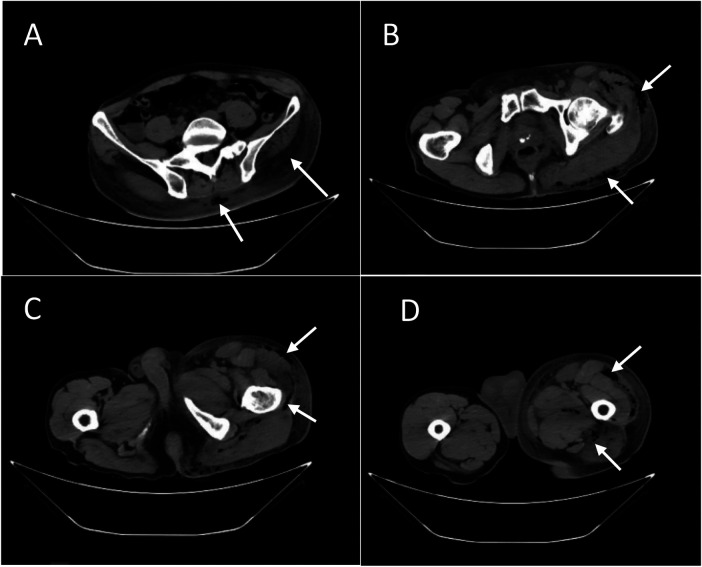
**(A–D)** the CT images obtained at the time of the patient's hospital admission.

Laboratory investigations revealed a significant inflammatory response: C-reactive protein (CRP) 261.0 mg/L, white blood cell (WBC) count 12.8 × 10^9^ /L, and procalcitonin 5.120 ng/mL. The Laboratory Risk Indicator for Necrotizing Fasciitis (LRINEC) score was 7, indicating a high probability of necrotizing fasciitis. Computed tomography (CT) confirmed diffuse soft tissue swelling and pathognomonic gas-density lesions (emphysema) tracking through the gluteal subcutaneous tissues and extending along the fascial planes of the left hip, thigh, and calf (Figures 1A–D).

### 2.3 Therapeutic intervention and surgical course

**Figure 2:**
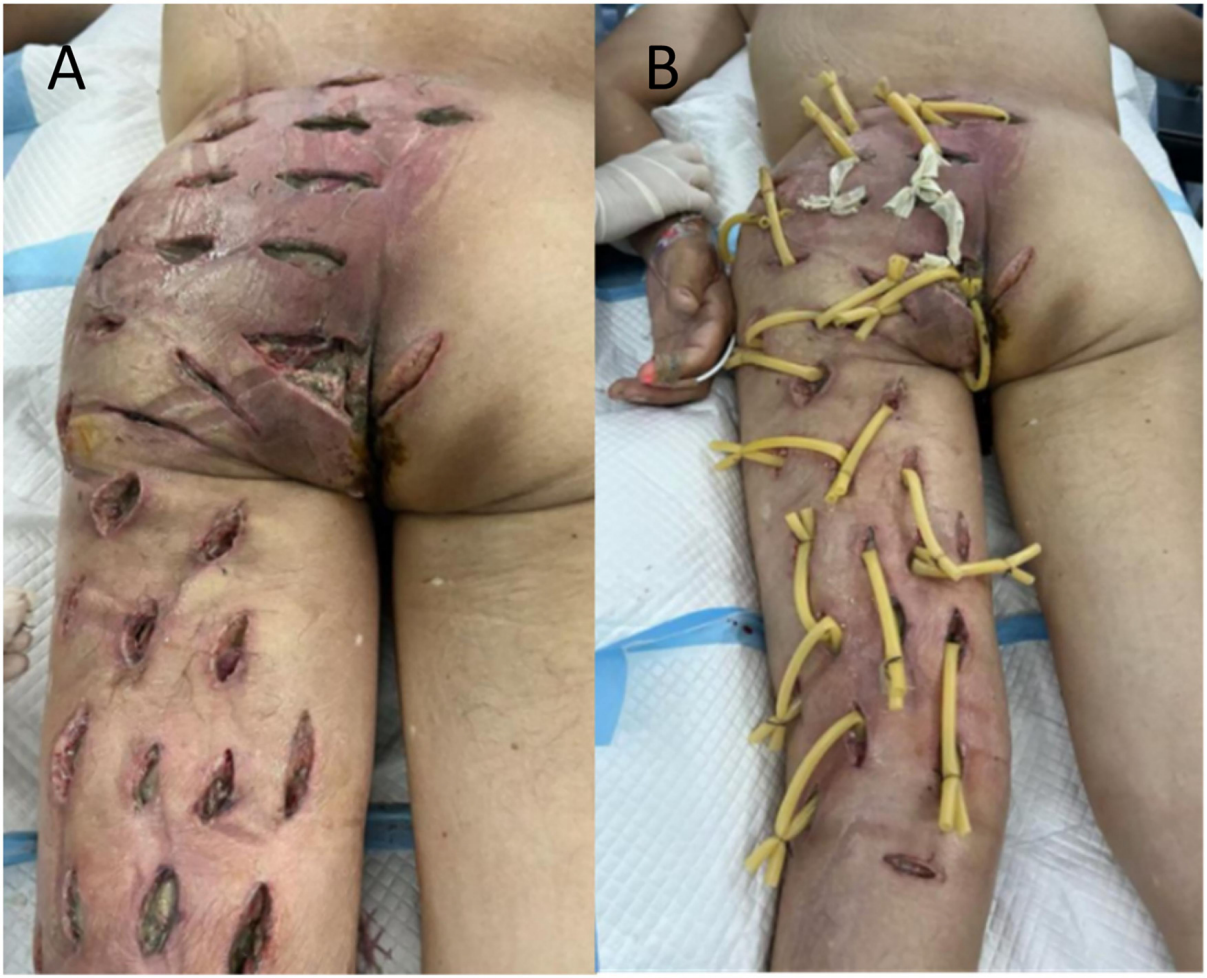
**(A,B)** photographs following the initial debridement.

The patient underwent emergency debridement on the night of admission. Intraoperatively, extensive fascial necrosis and malodorous pus were observed tracking from the perianal region to the mid-calf. The wound was meticulously irrigated with hydrogen peroxide, normal saline, and povidone-iodine until viable tissue was reached. To facilitate eventual closure, healthy skin bridges were preserved between incisions, and rubber drains were placed (Figures 2A,B). Postoperative wound cultures confirmed *Escherichia coli* infection, and systemic antibiotic therapy was escalated to imipenem-cilastatin.

**Figure 3:**
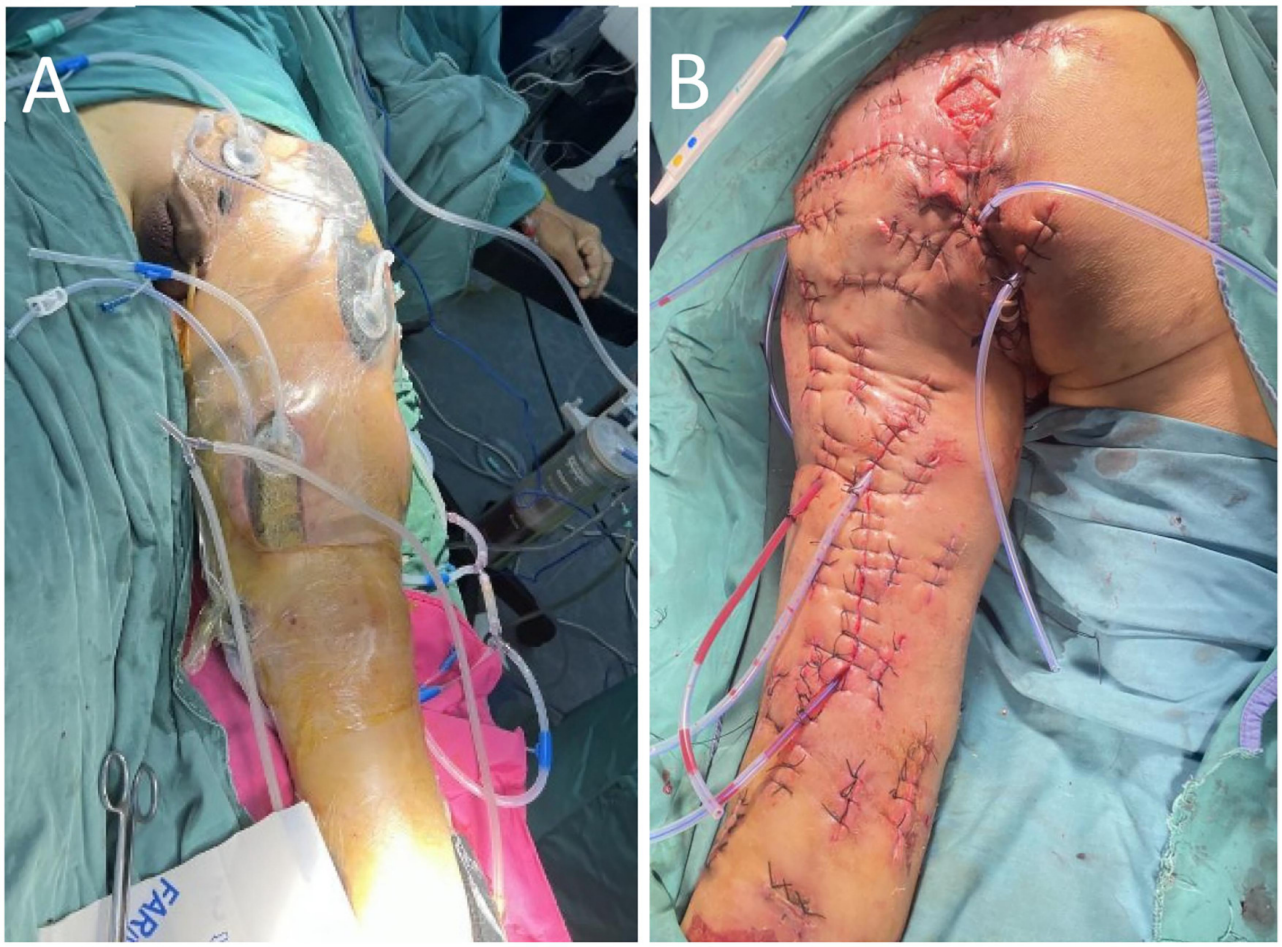
**(A)** following repeated debridement, the wound was managed with NPWT for irrigation and negative pressure drainage. **(B)** Upon completion of NPWT, the wound was sutured.

By postoperative day 3, however, clinical symptoms persisted, with continued malodorous drainage and crepitus. Repeat imaging and laboratory tests confirmed inadequate infection control. Consequently, a second debridement was performed on hospital day 4, immediately followed by the initiation of negative pressure wound therapy (NPWT). A vacuum-assisted closure (VAC) system was integrated with a bedside irrigation-suction setup, delivering 3,000 mL of normal saline daily through an irrigation tube placed deep within the wound (Figure 3A).

### 2.4 Outcome and follow-up

The patient's postoperative course remained stable, with inflammatory markers and metabolic parameters gradually normalizing. Negative pressure wound therapy (NPWT) was maintained through the third debridement on hospital day 9, at which point the wound beds exhibited complete clearance of necrotic debris and the emergence of healthy, exuberant granulation tissue. By the fourth procedure on day 11, the absence of residual necrotic tissue allowed for the initiation of wound reconstruction.

Owing to the efficacy of early source control, extensive skin loss was averted. The wound margins were meticulously debrided and primarily approximated under acceptable tension. Drains were positioned beneath each sutured incision, and NPWT was reapplied for an additional 48–72 h to facilitate continuous drainage and ensure optimal apposition of the wound surfaces.

**Figure 4:**
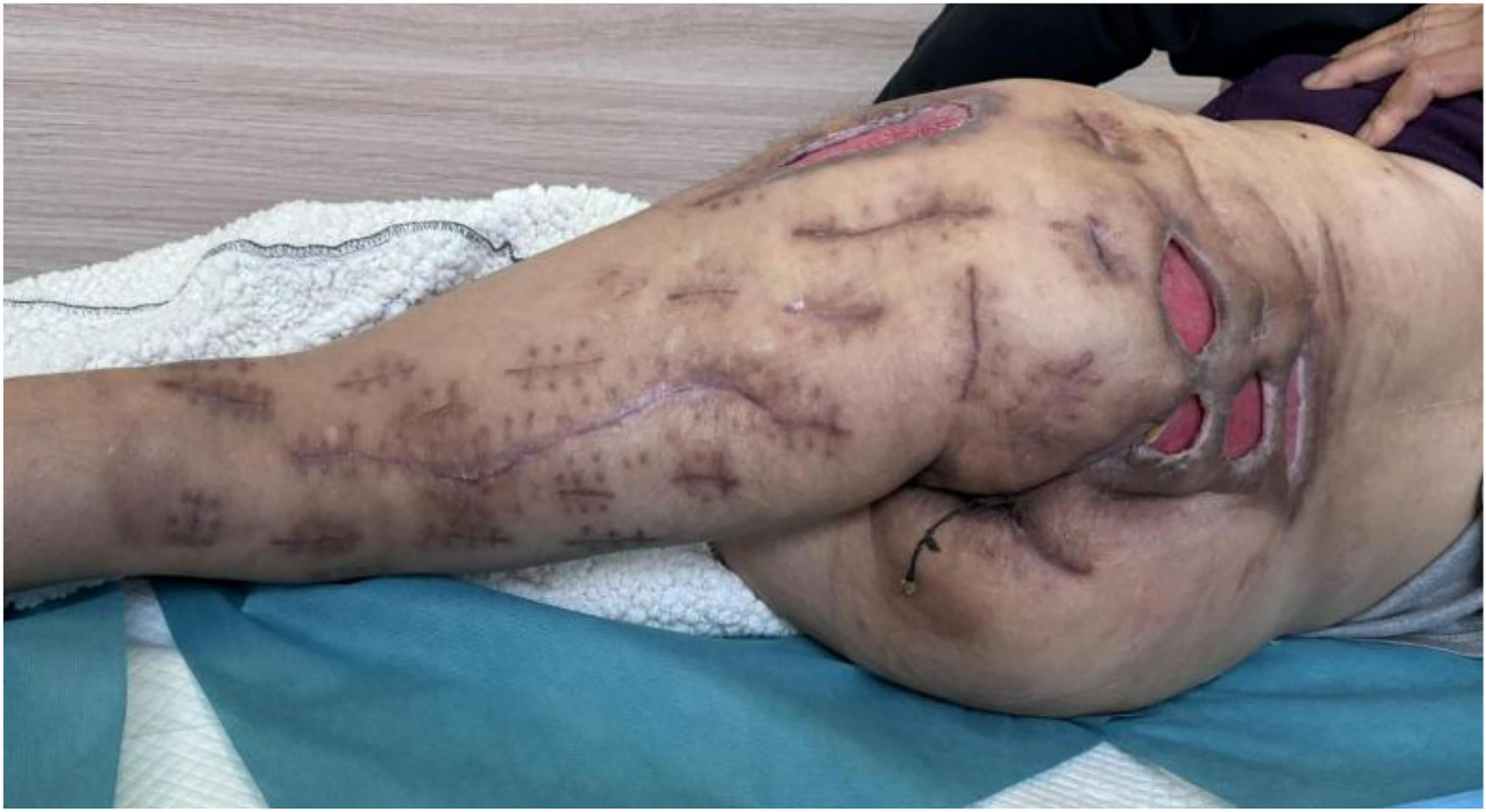
Photographs taken on postoperative day 37.

The majority of the incisions achieved delayed primary closure. Areas with significant skin defects or excessive tension were managed as open wounds with daily dressing changes (Figure 3B). These residual sites, supported by the preserved healthy skin bridges, healed successfully via secondary intention without the need for skin grafting. Final wound closure was performed on day 17, and the NPWT system was discontinued on day 19. Following a period of progressive suture removal and mobilized rehabilitation, the patient regained full ambulation and was discharged on hospital day 37 (Figure 4). At the one-year postoperative follow-up, the wounds remained stable and well-healed, with lower-limb motor function restored to pre-morbid levels.

## 3 Discussion

Perianal necrotizing fasciitis (PNF) remains a catastrophic surgical emergency characterized by precipitous progression and high mortality. Although the combination of early radical debridement and targeted antibiotic therapy constitutes the cornerstone of management (4), mortality rates remain stubbornly high. This persistence is partly attributable to the limitations of traditional, localized debridement and passive drainage techniques, which often fail to arrest the spread of infection through deep fascial planes.

### 3.1 Limitations of conventional surgery and the rationale for a modified approach

Standard techniques involving multiple small incisions and counter-drainage may suffice for localized infections. However, in cases where PNF tracks along deep fascial layers, these methods often leave “drainage blind spots,” predisposing the patient to refractory infection and sepsis. The extensive longitudinal debridement utilized in this case was designed to circumvent these issues. Its primary advantages are twofold: (1) it provides comprehensive exposure of the infected anatomy, facilitating meticulous debridement under direct visualization to minimize retained necrotic debris; and (2) it transforms a complex, infected space into an open, manageable wound bed. The surgical objective here shifts from merely “draining an abscess” to disrupting the anatomical pathways of infection and establishing a controllable wound environment.

### 3.2 The synergistic value and optimal timing of NPWT as a Key adjunctive therapy

The therapeutic efficacy of Negative Pressure Wound Therapy (NPWT) in managing PNF is well-documented (5–7). In the context of such fulminant infections, the therapeutic window is exceptionally narrow. Based on our clinical experience and a review of the literature, we propose that NPWT should be initiated immediately following the definitive diagnosis and the completion of the primary radical debridement. Early implementation facilitates rapid source control through continuous physical drainage, potentially circumscribing the infection's spread. This creates an optimal environment for subsequent interventions, potentially shortening the clinical course and mitigating patient morbidity.

However, the application of NPWT in the complex perineal region is frequently hampered by anatomical irregularities that complicate secure fixation and increase the risk of contamination. The success of this case was rooted in the systematic integration of NPWT following extended debridement. By utilizing the “clean” surgical foundation achieved through radical debridement, NPWT was able to effectively obliterate dead space and attenuate systemic toxin absorption.

Furthermore, the occlusive nature of the NPWT dressing served as a vital temporary barrier in an anatomically challenging zone, significantly decreasing the incidence of fecal contamination and markedly enhancing patient comfort (8). This integration establishes a synergistic model defined by “early radical debridement coupled with immediate NPWT support.” This dual-modality approach does more than merely accelerate granulation; it provides a stabilized local environment essential for definitive infection control. Our findings align with recent studies advocating for NPWT in the management of complex, infected abdominal and pelvic wounds (9).

### 3.3 Multidimensional supportive care and individualized fecal management

The favorable outcome was supported by a multidisciplinary team (MDT) approach, integrating intensive care, targeted antimicrobial therapy, and optimized nutrition. A pivotal point of discussion in PNF management is the role of prophylactic fecal diversion via colostomy (10). We advocate for an individualized strategy. While a diverting stoma may be necessary for patients with extreme contamination risk or anatomical barriers to NPWT fixation, our case demonstrates a viable stoma-sparing alternative. Through the use of indwelling rectal tubes and a strict elemental diet, we achieved effective fecal diversion without the morbidity of a stoma or the need for secondary reversal surgery. This approach, however, requires high-level perioperative vigilance and meticulous nursing care.

### 3.4 Insights, limitations, and future perspectives

This case suggests that for rapidly progressive and extensive PNF, the combination of thorough initial debridement and immediate NPWT application may be more decisive than traditional, staged conservative debridement. This integrated strategy facilitated rapid source control and mitigated the systemic inflammatory response, thereby significantly improving the patient's prognosis.

A notable observation in this case was that early containment of the infection limited the extent of skin and soft tissue necrosis. Furthermore, because necrotizing fasciitis primarily tracks along fascial planes, the underlying deep musculature remained viable. Consequently, the reconstruction phase did not require complex tissue transfers, such as fasciocutaneous flaps or muscle grafts. Instead, definitive closure was achieved through delayed primary suturing and direct approximation of the remaining healthy tissue—a result of the favorable wound environment created by the initial aggressive management.

Despite the successful outcome, several limitations must be acknowledged. First, as a single case report, this study lacks comparative data against established standard protocols; thus, the perceived superiority of this integrated approach requires validation through larger, prospective clinical trials. Second, the application of NPWT in the perineal and gluteal regions involves a specific technical learning curve and higher material costs, which may restrict its adoption in resource-limited clinical settings. Finally, while the one-year follow-up was positive, long-term outcomes regarding the quality of scar tissue and nuanced functional recovery warrant continued investigation.

For PNF patients presenting with more substantial dead spaces or massive tissue loss following debridement, more invasive reconstructive techniques—such as gracilis muscle flap interposition—have demonstrated potential in obliterating dead space and accelerating recovery (11). We advocate for an individualized treatment hierarchy based on the depth of the defect and the quality of the local tissue. The protocol of “radical debridement + early NPWT” presented here serves as a foundational framework. It stabilizes the patient and optimizes the local wound bed, providing clinicians with a “therapeutic bridge” to decide whether simple approximation is sufficient or if a transition to complex reconstructive procedures is necessitated.

## 4 Conclusions

In the context of delayed diagnosis and diffuse infection, the successful management of this PNF case was achieved through an integrated protocol of radical longitudinal debridement and early NPWT. The core value of this strategy lies in using aggressive surgery to create a wound bed optimized for vacuum-assisted drainage, thereby maximizing the biological benefits of NPWT. While technically demanding, this integrated framework offers a potent therapeutic rationale for treating extensive PNF and warrants further investigation in high-acuity surgical centers.

## Data Availability

The original contributions presented in the study are included in the article/Supplementary Material, further inquiries can be directed to the corresponding author.
